# Association of sleep quality with job burnout among Chinese coal mine staff: a propensity score weighting analysis

**DOI:** 10.1038/s41598-019-45329-2

**Published:** 2019-06-19

**Authors:** Xue Gao, Kai-Li Ma, Hui Wang, Qian Gao, Li-Jian Lei, Tong Wang

**Affiliations:** 1 Department of Health Statistics, School of Public Health, Shanxi Medical University, 56 Xinjiannanlu Street, Taiyuan, Shanxi 030001 China; 2 Department of Epidemiology, School of Public Health, Shanxi Medical University, 56 Xinjiannanlu Street, Taiyuan, Shanxi 030001 China

**Keywords:** Risk factors, Health occupations, Epidemiology

## Abstract

This study examines the association of sleep quality with job burnout among Chinese coal mine staff. 3832 subjects were selected from a coal mine group located in Shanxi Province in China. Job burnout was evaluated by the Maslach Burnout Inventory-General Survey and sleep quality was acquired with a self-reported questionnaire. We used the inverse probability of treatment weighting with propensity score to mimic the randomization and to minimize bias in estimations. Sensitivity analysis was conducted to test the robustness of our findings. We identified that good sleep quality was significantly associated with lower risk of job burnout (OR: 0.70; 95%CI, 0.60 to 0.82, p = 6.02e-06), with 0.21 decrease in the score of exhaustion (95%CI,−0.29 to −0.12, p = 5.00e-06), and with 0.13 decrease in the score of cynicism (95%CI,−0.21 to −0.04, p = 3.73e-03). Sensitivity analysis demonstrated that the results were robust to the choice of estimation models, as well as unmeasured confounding. Stratification analysis demonstrated that the associations of sleep quality with job burnout were largely heterogeneous for male and female workers. This study implicated that good sleep quality benefits the workers in relief of job burnout. Further research may be warranted in support of a definite causal relationship and intervention strategy.

## Introduction

Job burnout is a psychological syndrome as a mirror of long-term emotional and interpersonal stressors on the job^1,2^. Certain factors that may influence the experience of job burnout are both in terms of situational aspects and individual aspects^3–5^. One such factor could be sleep. Inferior sleep status can often lead to physical and mental exhaustion, such as cognitive performance, emotional well-being, work, and leisure activity, thus bring about adverse effects on physical health and mental health, including job burnout^6,7^.

On account of the special job characteristics and working conditions, coal mine workers are always much easily prone to be burnout than others^8^. Therefore, it is of great concern to explore the possible indicative factors of this special professional group to improve the personal psychological state and behavioral effect in their work. To be specific on the relationship of sleep-related status and job burnout, prior studies proposed mixed results^9–12^. It is not difficult to realize that the association of sleep status and job burnout differed among various kinds of workers. Different studies have been carried out on coal miners, but there has been no research specifically investigated the association of sleep and job burnout on this field of work^13,14^. So it is necessary to confirm the existence and magnitude of the potential relationship in this population for the consideration of occupational health.

For this reason, we sought to examine the association of sleep quality with job burnout on Chinese coal mine staff with propensity score method which can replicate a randomized experiment as closely as possible by obtaining balanced groups with similar covariate distributions^15^. The hypothesis to be confirmed is that there is a relationship between sleep quality and the experience of job burnout in coal mine workers.

Coal mine workers always need more emotional involvement because of the poor working conditions, excessive production demands, as well as the high incidence rate of occupational injury they exposed to. Such an occupational group is associated with significant psychosocial problems like job burnout^14^. The importance of this article is that it investigated the relationship between sleep quality and job burnout in coal miners for the first time, and thus identified a potential risk factor for job burnout in this occupational group for further research and intervention. Additionally, we stratified the association analyses by gender, age, and workplace, respectively, to derive more complete results specific to different subgroups. This study contributes to the previous researches which didn’t draw any conclusion about the relationship of sleep quality with job burnout among coal mine workers^13,14^, and provides a broader empirical basis for the relevant research on the mental health of coal miners.

## Material and Methods

### Study population

During the period of July 2013 to December 2013, we carried out a cross-sectional study in a large coal mine group in Shanxi province of China, which contained 87 coal mines and 200,000 permanent staff. With the sampling frame including age, gender and work type offered by the administrators of the coal mine group, we adopted two-stage cluster stratified sampling for the determination of the study sample. We estimated the sample size using the PASS version 11.0 (NCSS LLC: Kaysville, UT, USA)^16^. With the expected prevalence of burnout of 30.8%^17^, as well as an allowable error of 3.3%, a number of 3066 was calculated as the minimum sample size. Considering of no response and other uncertainties, we finally recruited 3832 subjects. Ten out of 87 coal mines were randomly selected as the primary sampling unit in the first stage, and 3832 subjects were extracted in the second stage^18^. We selected subjects with the inclusion criteria of being employed for at least one year, and exclusion criteria of (i) using neuroleptic drugs, tranquillizers or other drugs that may cause sleep alterations (ii) using antidepressants or anxiolytics, (iii) during pregnancy^19^. Among those being recruited, 10.41% of subjects were excluded because of missing information and a final total of 3433 coal mine staff remained.

The study had been approved by the Shanxi Medical University Ethics Committee. We performed the study in accordance with guidelines of the Declaration of Helsinki and we obtained the informed written consent from each participant.

### Maslach burnout inventory-general survey (MBI-GS)

Maslach Burnout Inventory-General Survey (MBI-GS) is the gold standard for measurement and identification of job burnout^1,20^, which is validated in a variety of occupations. The Chinese version of MBI-GS consists of 15 items with three dimensions describing different symptoms of burnout: exhaustion (EX, 5 items), which is the central reflection and the most prominent manifestation of burnout; cynicism(CY, 4 items), which reflects indifference, detached attitude towards work and active disengagement from work; and professional efficacy (PE, 6 items), which expresses the sense of competence and accomplishment during one’s work^20^. A standard 7-point Likert scale is used to measure the experience of job burnout with a score of each item ranging from 0(never) to 6(always), representing the frequency of the corresponding statement.

According to previous studies, the feeling of efficacy always appears simultaneously with overwhelming exhaustion and cynicism, thus to some extent, it can be substituted by the other two core dimensions^1,17,21^. For this reason, exhaustion and cynicism were selected in our research for the assessment of job burnout. Cronbach’s alpha was used for examining the reliability of the subscales, and the coefficients for EX and CY in our study were 0.910 and 0.880 respectively, indicating good internal consistency of the items in each subscale^22^. Additionally, we executed the confirmatory factor analysis using AMOS version 22.0 (IBM Corporation, Meadville, PA, USA) and calculated the validity of the subscales. The convergent validity for EX and CY were 0.664 and 0.646, respectively, indicating the appropriateness of the theoretical construct^23^.

### Outcome measures

The primary outcome variables were the prevalence of job burnout (dichotomous variable) and scores of the two dimensions: EX and CY (continuous variables). For the definition of job burnout, we utilized a widely accepted and applied standard to distinguish the clinical burnout from non-burnout individuals, i.e. either score >2.20 on the EX or score >2.00 on the CY as experiencing clinically job burnout^17,24–26^.

### Exposure variable and covariates

The exposure variable was sleep quality. By reference of the relevant research of sleep quality^27,28^, a self-administered questionnaire including eight items (range of each item score, 0–1): insufficient sleep, heavy snoring, use of sleep medication, subjective impaired sleep quality, premature awakening, troubles falling asleep, night waking frequently, waking up and then falling asleep difficultly, was used to measure the sleep quality. The sum of these 8 items yielded a global score, and higher scores represented more sleep complaints. Those with a global score of 0 were defined as healthy sleepers who had a good sleep quality, and a score higher than 0 represented sleep complainers who had a poorer sleep quality. The discrimination index of the questionnaire was 0.67 in our study, indicating its good performance in distinguishing healthy subjects from sleep-disordered subjects.

Demographic covariates included age, sex, marital status (married; unmarried; others), educational level (bachelor degree or above; junior college or below), current smoker and current drinker. Subjects who consumed at least one cigarette per day during the last month were defined as current smoker^29^, and who consumed alcoholic drinks at least once per month in the past year were defined as current drinker^30^.

Employees’ working conditions have an unneglectable influence on their wellbeing and efficiency on work, thus it is necessary to include the factors related to working conditions as covariates to control for the potential bias^31^. Working related covariates included work type (manual labor; mental labor), work seniority (1 to 3 years; 4 to 10 years; 11 to 15 years; 16 years or above), workplace (underground workers; ground workers), monthly income (4000 RMB or below; 4000 to 6000 RMB; 6000 to 8000 RMB; 8000 RMB or above), condition of work shifts and occupational injury. Shift work included working at any organization of hours that differ from the traditional diurnal work period^32^, and the occupational injury was defined as bodily damage resulting from working during the past three years^33^.

### The propensity score methods

Being the optimal approach of determining the association between potential risk factor and outcome, randomized clinical trials (RCTs) would not be feasible in our study because it was impractical to randomize the allocation of the subjects based on their sleep quality^34,35^. Propensity score (PS) analysis could address this issue by assigning to each subject a propensity score defined as the likelihood of being exposed to an event of interested given a set of baseline covariates. Conditional on this score, covariates would be distributed equally in different groups^36–38^. PS methods are broadly used in medical studies with observational design, with the advantages of allowing observational studies to be designed similar to randomized experiments and making exposure status independent of the distribution of baseline covariates^36,39–41^. Compared with traditional multivariable adjustment methods, PS methods allow a more straightforward presentation of whether the balance of confounders was achieved in different groups^42^. Additionally, this approach actually allows less biased estimates than the multivariate regression when the covariates are of extreme imbalance in the exposed and controlled groups^43–45^.

### Statistical analysis

First of all, we checked the balance of covariates between exposure and control groups by calculating the standardized effect size for each covariate. Bigger values of standardized effect size predicted severer imbalance, and a value bigger than 0.1 indicated statistically significant non-match of covariate distributions^46^.

We then accounted for the bias due to the difference between exposed subjects and healthy controls. To achieve this, we estimated the propensity score and weighted the subjects using the propensity score. Firstly we implemented propensity score estimations with the generalized boosted model (GBM), which showed robust properties and low prediction error among various PS estimation methods^47,48^. The exposure variable (good sleep quality vs. poorer sleep quality) was regressed on the above covariates in PS estimation model. Then we utilized the inverse probability of treatment weighting (IPTW) using propensity score for the sample weighting process, with which we could achieve the comparability between exposed and controlled groups^49,50^. To assess whether resulting propensity score weights had removed the imbalance between different groups, we calculated the standardized effect size to check the quality of balancing performance after the weighting process^51^.

The next step was to estimate the association of sleep quality and job burnout with the weighted sample. We investigated the association with the logistic model for the categorical outcome and linear models for the continuous outcomes.

### Sensitivity analysis

To help increase the confidence of our findings, we conducted a series of sensitivity analyses. Firstly, to assess the sensitivity of results to the choice of PS estimation models, we considered estimating the propensity scores using logistic regression instead of the generalized boosted model^52^. Secondly, to assess the sensitivity of results to the choice of outcome estimation models, we executed traditional covariate adjustment regression and compared the results with PS weighting regression results in order to acquire the doubly robust effect estimators^53^. Thirdly, to assess the sensitivity of results to unmeasured confounding, we estimated the quasi “true association” between exposure and the outcome adjusting for the hypothetical unobserved confounder and compare the estimators with the results derived from our study^44,52,54^.

### Stratification analysis

Finally, we repeated the PS estimation models in subgroups stratified by gender(male/female), age (≤40 years old/>40 years old) and workplace (unground workers/ground workers) severally, for the purpose of further examining whether the associations of sleep quality with job burnout varied in different subgroups, and of summarizing extensive and objective policy lessons from the corresponding results.

All the tests were evaluated at 2-sided with a p-value of less than a 0.05 considered as statistically significant. Data were collected and analyzed using Epi info version 3.5.1 (CDC, Atlanta, GA, USA), and R version 3.5.0 (http://cran.r-project.org).

## Results

### Sample characteristics

Table 1 illustrated the baseline characteristics of the original sample. Among 3433 participants, 1327(38.65%) reported good sleep quality and 2106 (61.35%) reported poorer sleep quality. The subjects with poorer sleep quality were more likely to be smokers and drinkers, while there were more workers who didn’t have nightshifts on work in the group of good sleep quality. As displayed in Table 1, before weighting the sample, substantial differences were observed across two groups on most of the covariates. Robust weighting approaches are therefore needed to limit the extent of confounding bias.

**Table 1:** Sample characteristics.

	Total	Good sleep quality	Poorer sleep quality	d^a^
Age (Mean ± SD)	41.03 ± 8.64	40.10 ± 8.67	41.62 ± 8.57	−0.176
Male sex (%)	84.6	81.7	86.40	−0.130
**Marital status (%)**				
married	93.6	93.1	94.0	−0.036
unmarried	4.8	6.0	4.0	0.081
others	1.6	1.0	2.0	−0.103
Bachelor degree/above (%)	12.9	13.7	12.3	0.042
Current smoking (%)	57.8	53.4	60.5	−0.146
Alcohol consumption (%)	41.4	35.6	45.1	−0.194
Manual labor (%)	74.1	73.9	74.3	−0.011
**Work seniority (year) (%)**				
1–3	12.6	15.1	11.1	0.114
4–10	23.9	25.8	22.7	0.069
11–15	16.5	16.4	16.5	−0.003
≥16	47.0	42.7	49.7	−0.142
Underground workers (%)	55.9	52.6	58.0	−0.109
**Monthly income (%)**				
≤4000	24.8	22.1	26.4	−0.105
4000–6000	42.7	41.4	43.5	−0.042
6000–8000	22.5	25.4	20.8	0.107
≥8000	10.0	11.1	9.3	0.056
Work shifts (%)	48.3	39.5	53.8	−0.287
Occupational injury (%)	9.8	8.3	10.7	−0.082

Abbreviations: SD, standard deviation.

^a^Standardized effect size.

### Diagnostics of IPTW using propensity score

After weighting the subjects with inverse probability using the propensity scores, differences between groups diminished substantially. The standardized effect sizes were less than 0.05 for all the covariates, as would be expected in the test for covariate differences in a random experiment. There was no longer any difference between the two groups after the weighting process, thus validating the good balance of subjects after the execution of weighting (Table 2).

**Table 2:** Inverse probability weighted-sample characteristics.

	Good sleep quality	Poorer sleep quality	d^a^
Age (Mean ± SD)	40.97 ± 8.51	41.09 ± 8.59	−0.014
Male sex (%)	84.4	84.6	−0.006
**Marital status (%)**			
married	94.3	93.9	0.016
unmarried	4.5	4.5	0.003
others	1.2	1.6	−0.041
Bachelor degree/above (%)	12.7	13.0	−0.010
Current smoking (%)	57.2	58.0	−0.017
Alcohol consumption (%)	40.8	41.8	−0.020
Manual labor (%)	74.9	74.1	0.019
**Work seniority (year) (%)**			
1–3	12.6	12.0	0.016
4–10	24.4	24.2	0.004
11–15	16.5	16.3	0.004
≥16	46.6	47.4	−0.017
Underground workers (%)	55.8	56.5	−0.014
**Monthly income (%)**			
≤4000	24.3	25.0	−0.016
4000–6000	42.1	42.4	−0.007
6000–8000	23.7	22.5	0.027
≥8000	10.0	10.0	−0.003
Work shifts (%)	47.4	48.7	−0.026
Occupational injury (%)	9.3	9.9	−0.018

Abbreviations: SD, standard deviation.

^a^Standardized effect size.

### Prevalence of job burnout and other outcomes

Overall, the total prevalence of job burnout was 35.48% among the participants, and the average scores of EX and CY were 1.74 and 1.29, respectively. No additional covariates were needed to adjust in the next PS regression model because the weights had accounted for significant differences across different groups^15^. With the generalized boosted PS weighting regression model, we identified that good sleepers were significantly associated with lower risk in the odds of exposure to job burnout, in comparison with poorer sleepers (OR: 0.70; 95%CI, 0.60 to 0.82, p = 6.02e-06). In addition, good sleep quality was significantly associated with 0.21 lower in the score of EX (95%CI, −0.29 to −0.12, p = 5.00e-06) and 0.13 lower in the score of CY (95%CI, −0.21 to −0.04, p = 3.73e-03) (Table 3).

**Table 3:** Models examining the association of sleep quality with job burnout.

	GBM-based PS weighting model^*^		
	Estimate (SE)	p value	95%CI
Job burnout (OR)^a^	0.70 (0.08)	6.02e-06	(0.60, 0.82)
EX^b^	−0.21 (0.05)	5.00e-06	(−0.29, −0.12)
CY^c^	−0.13 (0.04)	3.73e-03	(−0.21, −0.04)

^*^Regression with the weighted sample by inverse probability of treatment weighting method, with propensity score derived from the generalized boosted model.

^a^The association of sleep quality with job burnout by logistic regression.

^b^The association of sleep quality with exhaustion by linear regression.

^c^The association of sleep quality with cynicism by linear regression.

Abbreviations: GBM: generalized boosted model, SE, standard error, CI: confidence interval, OR: odds ratio, EX: exhaustion, CY: cynicism.

### Sensitivity analysis

With the logistic-based PS weighting regression model, we would declare a significant association of sleep quality with job burnout. Subjects with good sleep quality showed lower risk to be burnout in comparison to poorer sleepers (OR: 0.69; 95%CI, 0.59 to 0.81, p = 4.56e-06). Good sleep quality significantly decreased the scores by 0.20 in EX (95%CI, −0.29 to −0.12, p = 6.47e-06) and 0.12 in CY (95%CI, −0.21 to −0.03, p = 6.13e-03) more than poorer sleep quality. (Table 4, model 1) The estimators using covariate adjusting regression model were also similar to the above two PS models. (Table 4, model 2) In addition, we quantified the unmeasured confounder which is related to both exposure and the outcome under some certain specifications according to Lin’s approach, and estimated the association adjusting for the above-unmeasured confounder, together with all measured confounders. After adjustment, the estimated quasi “true effects” were all quite similar to the results of our methods (Table 4, model 3).

**Table 4:** Sensitivity analysis.

	Logistic-based PS weighting model^*^			Covariate adjusting regression model^**^			Unmeasured confounding adjusting model^†^	
	Estimate(SE)	p value	95%CI	Estimate(SE)	p value	95%CI	Estimate	95%CI
Job burnout(OR)^a^	0.69 (0.08)	4.56e-06	(0.59, 0.81)	0.69 (0.08)	5.42e-06	(0.59, 0.81)	0.69	(0.59, 0.81)
EX^b^	−0.20 (0.05)	6.47e-06	(−0.29, −0.12)	−0.20 (0.04)	5.28e-06	(−0.28, −0.11)	−0.20	(−0.28, −0.11)
CY^c^	−0.12 (0.04)	6.13e-03	(−0.21, −0.03)	−0.11 (0.04)	0.01	(−0.20, −0.03)	−0.11	(−0.20, −0.03)

^*^Regression with the weighted sample by inverse probability of treatment weighting method, with propensity score derived from the logistic regression model.

^**^Covariate adjusting regression model adjusting for covariates in Table 1.

^†^Estimation of “true effect” adjusting quantified unmeasured confounding.

^a^The association of sleep quality with job burnout by logistic regression.

^b^The association of sleep quality with exhaustion by linear regression.

^c^The association of sleep quality with cynicism by linear regression.

Abbreviations: PS: propensity score, SE, standard error, CI: confidence interval, OR: odds ratio, EX: exhaustion, CY: cynicism.

The homogeneity of GBM-based PS model and logistic-based PS model showed no evidence of misspecification of the PS estimation model. The homogeneity of the PS regression model and traditional covariate adjusting regression model guaranteed the double robustness of our findings. The results were not sensitive to the choice of PS-estimation models and the choice of outcome-estimation models. Moreover, the homogeneity of the unmeasured confounding adjustment model and PS regression models suggested that the study inference was not sensitive to unobserved confounding. Therefore, we could conclude from the above sensitivity analysis that the results were robust and reliable.

### Stratification analysis

At last, we repeated all the PS estimation models in subgroups stratified by age, gender and workplace. Heterogeneity existed in the relationships between different stratifications. In the analysis stratified by age, we found significant association of sleep quality with job burnout (OR: 0.66; 95%CI, 0.53 to 0.83, p = 3.49e-04), as well as with EX (Beta = −0.26, 95%CI, −0.38 to −0.14, p = 2.64e-05) and CY (Beta = −0.15, 95%CI, −0.27 to −0.02, p = 0.02) in the youth workers. In the middle-aged worker group, the association of sleep quality with job burnout (OR: 0.70; 95%CI, 0.57 to 0.87, p = 1.55e-03) and with EX (Beta = −0.17, 95%CI, −0.30 to −0.04, p = 0.01) were still statistically significant. However, there was no longer a significant association of sleep quality with CY in this group (Beta = −0.11, 95%CI, −0.23 to 0.01, p = 0.08). The condition was the same in the analyses stratified by the workplace. For the underground workers, significant associations were observed in the sleep quality with three measures of job burnout; for the ground workers, however, sleep quality was significantly associated with job burnout and EX but was unassociated with CY any longer (Table 5).

**Table 5:** Subgroup analysis examining the association of sleep quality with job burnout.

Stratification			GBM-based PS weighting model^*^		
			Estimate(SE)	p value	95%CI
Age	≤40 (47.1%)	Job burnout (OR)^a^	0.66 (0.11)	3.49e-04	(0.53, 0.83)
		EX^b^	−0.26 (0.06)	2.64e-05	(−0.38, −0.14)
		CY^c^	−0.15 (0.06)	0.02	(−0.27, −0.02)
	>40 (52.9%)	Job burnout (OR)^a^	0.70 (0.11)	1.55e-03	(0.57, 0.87)
		EX^b^	−0.17 (0.07)	0.01	(−0.30,−0.04)
		CY^c^	−0.11 (0.06)	0.08	(−0.23,0.01)
Gender	Male (84.6%)	Job burnout (OR)^a^	0.66 (0.08)	4.94e-07	(0.56,0.77)
		EX^b^	−0.25 (0.05)	6.25e-07	(−0.35,−0.15)
		CY^c^	−0.16 (0.05)	7.56e-04	(−0.25,−0.07)
	Female (15.4%)	Job burnout (OR)^a^	0.99 (0.27)	0.97	(0.58,1.68)
		EX^b^	0.05 (0.09)	0.56	(−0.12,0.22)
		CY^c^	0.07 (0.09)	0.45	(−0.11,0.24)
Workplace	Underground (55.9%)	Job burnout (OR)^a^	0.66 (0.10)	5.06e-05	(0.54,0.80)
		EX^b^	−0.27 (0.06)	1.98e-05	(−0.40,−0.15)
		CY^c^	−0.20 (0.06)	1.34e-03	(−0.32,−0.08)
	Ground (44.1%)	Job burnout (OR)^a^	0.74 (0.13)	0.02	(0.58,0.95)
		EX^b^	−0.12 (0.06)	0.03	(−0.24,−0.01)
		CY^c^	−0.03 (0.06)	0.58	(−0.15,0.09)

^*^Regression with the weighted sample by inverse probability of treatment weighting method, with propensity score derived from the generalized boosted model.

^a^The association of sleep quality with job burnout by logistic regression.

^b^The association of sleep quality with exhaustion by linear regression.

^c^The association of sleep quality with cynicism by linear regression.

Abbreviations: GBM: generalized boosted model, SE, standard error, CI: confidence interval, OR: odds ratio, EX: exhaustion, CY: cynicism.

We recognized that the results that are substantially inconsistent in two gender groups. In the male group, we obtained that good sleep quality was significantly associated with lower risk of burnout (OR: 0.66; 95%CI, 0.56 to 0.77, p = 4.94e-07), with a 0.25 decreased in the score of EX (95%CI, −0.35 to −0.15, p = 6.25e-07) and with a 0.16 decreased in the score of CY (95%CI, −0.25 to −0.07, p = 7.56e-04). However, sleep quality was not associated with a significant difference in the risk of burnout and scores of two dimensions of burnout in the female group (Table 5).

## Discussion

Based on the observational study, we identified a strong association of sleep quality with job burnout on coal miners in China. According to the results, good sleep quality was significantly associated with thirty percent lower in the risk of job burnout (OR: 0.70; 95%CI, 0.60 to 0.82, p = 6.02e-06). Workers with good sleep quality were less likely to experience job burnout compared with those with bad sleep quality, indicating that good sleep quality is beneficial to the alleviation of the syndrome of job burnout. Meanwhile, good sleep quality was significantly associated with 0.21 lower in the score of EX (95%CI, −0.29 to −0.12, p = 5.00e-06) and 0.13 lower in the score of CY (95%CI, −0.21 to −0.04, p = 3.73e-03). Exhaustion is predictive of the stress-related dimension of burnout, and cynicism represents the interpersonal context dimension of burnout^1^. Thus, significant relationships of sleep quality with these two aspects of job burnout indicated that good sleep quality may alleviate the stress and facilitate the emotional experience in their work. The findings could provide implications for coal mine workers regarding potential intervention targets. In order to relieve the experience of job burnout, improvement of sleep quality seems to be a link that cannot be neglected.

The study subjects we chose are engaged in coal mine industry, which is a special occupation. They are always faced with tough working conditions, which impair workers’ well-being physiologically and psychologically. In this survey study, male workers make up the majority of the whole sample (84.6%). The condition is similar for the less educated workers, which occupied 87.1% of the whole sample. Moreover, 74.1% of the workers are engaged in manual labor, and 55.9% of the workers worked in the underground of the coal mine. These characteristics further illustrate the difference between coal miners and other types of workers.

Through the stratification analysis, we realized that the associations of sleep quality with job burnout differ in various subgroups. Good sleep quality contributed more to the decrease in the risk of job burnout among the youth workers (OR = 0.66, 95%CI, 0.53 to 0.83, p = 3.49e-04) than that among the middle-aged workers (OR = 0.70, 95%CI, 0.57 to 0.87, p = 1.55e-03). Similarly, good sleep quality contributed more to the decrease in the risk of job burnout among the underground workers (OR = 0.66, 95%CI, 0.54 to 0.80, p = 5.06e-05) than that among the ground workers (OR = 0.74, 95%CI, 0.58 to 0.95, p = 0.02). These results suggested that for workers that are under different conditions, sleep quality predicts differently for job burnout. Specifically, for our findings, it seemed that an identical intervention for sleep quality could benefit more for the youth workers than that for the middle-aged workers, similarly for the underground workers than that for the ground workers, in terms of job burnout. However, we didn’t observe the association of sleep quality with any measure of job burnout among female workers. The insignificant results for female workers were probably caused by the insufficient sample size and power for the estimations, basing on the fact that the proportion of female workers was much smaller than that of male workers in our sample.

The mechanism behind burnout is complicated, but there are several findings in clinical practice providing relevant evidence for the association discovered in our study. As a direct reflection of sleep quality, clinical sleep disturbances may result in a later occurrence of job burnout^11^. More explicitly, research yielded evidence supporting the mechanism linking job burnout with sleep disturbances through the dysregulation of the hypothalamic–pituitary–adrenal axis along with sympathetic nervous system activation^55,56^.

As for the relationship between sleep-related status and job burnout, there were several relevant studies that proposed mixed results with limited generalization. Ekstedt and Söderström implemented studies on white-collar workers and demonstrated that impaired sleep may play a role in the development of fatigue or exhaustion in burnout^9^, and recovery from burnout is often accompanied by increased sleep continuity^12^. They also conducted a study on employees in a technology company and found that young subjects with high burnout scores suffer more sleep problem during days off^11^. However, Rosen IM carried out a study among interns in the internal medicine resident program at the University of Pennsylvania and found no significant associations between the development of chronic sleep deprivation and any of the burnout subscales^10^.

It is obvious to know from prior studies that the associations of sleep status with job burnout varied in terms of the existence and magnitude among different kinds of workers. Coal mine staffs are apt to sleep problems and job burnout owing to the special work pattern, while relevant studies that especially aimed at industrial workers are quite scarce. We can hardly find evidence of that association among workers in such fields. Thus, we focused our study on this type of workers and uncovered the strong association of sleep quality with job burnout in this population. What’s more, there was no conclusion about the association of integrated sleep quality and job burnout in prior studies. For this consideration, we measured the global sleep quality containing eight aspects of sleep status and demonstrated an explicit result with respect to the association.

Estimation relying on observational research is invariably subject to confounding, thus brought about selection bias and misleading results^35,57^. To solve the potential problems, we chose the inverse probability of treatment weighting using the PS method to equate the confounding and thereby facilitate the outcome model. A number of authors have shown that methods such as linear regression adjustment can actually increase bias in the estimated treatment effect when there are large differences of the covariates between the treated and control groups^39,58–60^. Nevertheless, PS models can address this matter by establishing an artificial population in which baseline covariates are independent of treatment status and different groups are comparable. The imbalance is bound to happen in the non-experimental study, so we preferred the optimum weighting methods with PS to remove the bias in the study.

However, our study should be interpreted in the context of the following limitations. The unmeasured confounding is the Achilles heel of most observational studies^61^. For example, individuals’ psychiatric conditions are likely to correlate with sleep status and job burnout. Not accounting for this sort of covariates may bias the estimations. To address this problem to the maximum extent, we adopted a series of concrete steps. In the data collection procedure, we excluded the subjects who are taking medication for treating mental illness. That is to say, we ruled out the influence of psychiatric disorders and related drug effects on participants’ sleeping or psychological status. Furthermore, sufficient prior knowledge was applied in designing the study and analyzing the data. We collected both demographic and work-related factors and included them in the PS model to account for confounding associated with both the exposure and outcome variables. In the analytical process, we implemented the sensitivity analysis and proved that the results were not sensitive to unmeasured confounder. These measures might help increase confidence in results from nonexperimental studies. However, the assumption of no unmeasured confounding can never be proven for certain.

Besides, with the utilization of the cross-sectional design, we could never acquire a causal relationship. With the PS models being used, we methodologically obtained a robust estimation of the association. This may give assistance to establish the evidence of the causal argument but does not prove it any further. Thus, further studies may be warranted in support of a definite causal relationship.

## Conclusions

With a large-scale survey on Chinese coal mine staff, we observed that sleep quality is significantly associated with various aspects of job burnout. In particular, good sleep quality is strongly associated with a lower risk of job burnout. Good sleep quality is also associated with two different symptoms of burnout: exhaustion and cynicism. When estimating the relationships in subgroups, the results showed that in terms of relieving job burnout, good sleep quality benefits more for the youth workers than the middle-aged workers, as well for the underground workers than the ground workers.

Good sleep quality had a positive hint on the suppression of the prevalence of job burnout and on the alleviation of clinical symptoms of job burnout. These findings filled in the knowledge gap about the relationship between sleep quality and job burnout in this special professional population and offered the coal mine workers a credible proof for deeply understanding about their psychological states. In the meantime, the study realized a complement for the previous research about the exploration of the mental health status of coal miners.

Individual’s sleep quality is much easier to assess and improve than other uncontrollable factors related to job burnout, thus our findings presented a practical significance for occupational and public health. Effective interventions to enhance sleep could help to prevent job burnout, and regain wellbeing in their occupational life. More research is needed in the future to explore the causality between sleep and job burnout and the intervention strategies regarding public and occupational health perspective.

## Data Availability

The datasets generated during and/or analysed during the current study are available from the corresponding author on reasonable request.
